# Spatial and Temporal Activity Patterns of the Free-Living Giant Mole-Rat (*Fukomys mechowii*), the Largest Social Bathyergid

**DOI:** 10.1371/journal.pone.0055357

**Published:** 2013-01-30

**Authors:** Matěj Lövy, Jan Šklíba, Radim Šumbera

**Affiliations:** Department of Zoology, Faculty of Science, University of South Bohemia, České Budějovice, Czech Republic; University of Arizona, United States of America

## Abstract

Despite the considerable attention devoted to the biology of social species of African mole-rats (Bathyergidae, Rodentia), knowledge is lacking about their behaviour under natural conditions. We studied activity of the largest social bathyergid, the giant mole-rat *Fukomys mechowii*, in its natural habitat in Zambia using radio-telemetry. We radio-tracked six individuals during three continuous 72-h sessions. Five of these individuals, including a breeding male, belonged to a single family group; the remaining female was probably a solitary disperser. The non-breeders of the family were active (i.e. outside the nest) 5.8 hours per 24h-day with the activity split into 6.5 short bouts. The activity was more concentrated in the night hours, when the animals also travelled longer distances from the nest. The breeding male spent only 3.2 hours per day outside the nest, utilizing less than 20% of the whole family home range. The dispersing female displayed a much different activity pattern than the family members. Her 8.0 hours of outside-nest activity per day were split into 4.6 bouts which were twice as long as in the family non-breeders. Her activity peak in the late afternoon coincided with the temperature maximum in the depth of 10 cm (roughly the depth of the foraging tunnels). Our results suggest that the breeding individuals (at least males) contribute very little to the work of the family group. Nevertheless, the amount of an individual's activity and its daily pattern are probably flexible in this species and can be modified in response to actual environmental and social conditions.

## Introduction

The subterranean ecotope provides its mammalian inhabitants with safety from predators and stable micro-environmental conditions. On the other hand, this environment is permanently dark and short of most sensory cues relevant for timing of activity and for spatial orientation [1]. Because of the hidden way of life of subterranean mammals, there is only limited knowledge on their spatial and temporal activity patterns under natural condition. This situation is in contrast with more numerous studies on the activity of captive subterranean mammals, most of which are focused on circadian activity rhythms and the role of light in their entrainment ([2]–[5] and citations therein).

Among subterranean mammals, African mole-rats (Bathyergidae, Rodentia) are an excellent subject for studying how various factors can influence activity patterns. First, this family contains strictly subterranean species that rarely come out of their burrow systems. This would assume that factors other than light might determine their activity patterns under natural conditions. One of the potential factors is underground temperature. Its daily fluctuations were proposed to determine the activity pattern in solitary *Heliophobius argenteocinereus* [4]. Second, this family provides a good opportunity to test how an activity pattern can be affected by social factors. Most bathyergids are social, living in multigenerational family groups [6], which is unique among subterranean mammals [1]. The groups usually utilize a communal nest [7], which would assume that the activity of the individuals could be triggered also by social cues (cf. [8], [9]) possibly resulting in more evenly distributed activity across the 24 h day. Available data to test this assumption are, nevertheless, very sparse (cf. [10]).

The amount of activity performed by individuals of a captive mole-rat family group usually varies. Breeders and larger non-breeders usually perform less activity than smaller non-breeders (e.g. [11], [12]), but several exceptions were reported (e.g. [13], [14]). To better understand this phenomenon, more data are necessary from free-living mole-rat family groups.

A convincing method to reveal differences between individuals' activity patterns within an animal group in the field is radio-telemetry. In free-living social bathyergids, this method was used so far only twice. Lovegrove [10] radio-tracked five individuals of *F. damarensis*. These animals spent only 24% of their time outside the nest and their daily activity patterns were irregular, with on average six short bouts of activity per day and no conspicuous differences between the individuals. The second radio-telemetry study was carried out on *Heterocephalus glaber* but it brought no data on individuals' activity patterns [15].

In the present study we analyse activity of the giant mole-rat *Fukomys mechowii*, the largest social species of African mole-rats. Giant mole-rats live in family groups of around ten individuals [16], [17] which typically consist of a single breeding pair and its non-breeding offspring. As in other social mole-rats, the family members are supposed to cooperate in energetically costly work tasks such as excavation of new tunnels in order to locate food resources (underground storage organs of plants), carrying food items into food stores and pushing excavated soil into aboveground mounds or into older unused tunnels of their burrow system [7]. To reveal spatial and temporal activity patterns in this species, inter-individual variability of these patterns and their possible determinants, we radio-tracked six individuals (five of them, including a breeding male, were from the same family group). The specific objectives of our study were (a) to compare space-use of individuals belonging to a single family; (b) to compare daily activity patterns of the breeding male with the patterns of non-breeding individuals; and (c) to relate the individuals' activity patterns to daily cycles of temperature in various depths of soil and with the natural cycle of daylight.

## Materials and Methods

### Ethic statement

All procedures involving wild-caught animals were performed in a human manner and were approved by the Zambia Wildlife Authority (ZAWA) and the Institutional Animal Care and Use Committee at University of South Bohemia and Ministry of Education, Youth and Sports (n. 12935/2007/30). The official permit n. 223623 approving the field research on the giant mole-rat in Zambia was issued by ZAWA. After the end of the study, radio-collared animals were recaptured and they were transported alive to the University of Duisburg-Essen for further research purposes.

### Study species

The giant mole-rat (*Fukomys mechowii*) is the largest social mole-rat weighing 345±95 (250–560) g in adult males and 252±34 (200–295) g in adult females [18]. The area of their distribution (Democratic republic of Congo, Zambia and Angola) is generally mesic, corresponding to the precipitation isoline of 900 mm of annual rainfall [19].

### Study site

The study was conducted from April to June 2009 at the Ndola Hill Forest Reserve, Copperbelt Province, Zambia (12°58′ S, 28°35′ E; 1290 m a. s. l.). The study area is covered by natural Miombo woodland. The climate in Zambia is characterized by a rainy season (October/November – March/April), a dry and cold season (April – July) and a dry and hot season (August – October). There was no precipitation in the course of the study.

### Field works

Mole-rats were captured at two trapping sites 250 m apart (in burrow systems of two different family groups) using the Hickman live-traps set into tunnels uncovered near fresh mole-hills. One adult female (F202, weighing 220 g) was captured at site 1. Since the efficiency of the traps was low, at site 2 we combined the traps with a partial excavation of deep tunnels around an accidentally found nest. Here, we captured a family of 11 individuals: eight females (33, 90, 106, 108, 166, 192, 207 and 208 g) and three males (221, 231 and 454 g). The largest male had massive head muscularity, pigmented corners of the mouth and a conspicuously large penis and testes. All other family members displayed conspicuously submissive behaviour towards him. We therefore suppose that he was the breeding male of the family. No breeding female (female with enlarged teats and perforate vagina) was captured. All males and 2 females (192 g and 207 g) from site 2 and the female from site 1 were shortly anesthetized by ketamine and xylazine and fitted with radio-collars (Brass collar, Pip transmitter with a position-based activity indicator; Biotrack Ltd, Dorset, UK). The weight of the radio-collars was less than 5% of the body mass of the smallest radio-tracked animal under study. The activity indicator changed the signal rate of the transmitter when the collar departed from a vertical position (e.g. when the animal lowered its head to a curled-up position, as when sleeping), which was used to identify the body position of the animals encountered inside the nest. All animals were released into burrows at their capture sites within 72 hours after their capture. Radio-tracking started 20 days after the release of the last animals. At this time the animals of site 2 already used a communal nest newly built at the same place where the original one had been destroyed, and it was detected that they freely move across the previously damaged area by re-established tunnel connections. Female 202 left site 1 shortly (between 7 and 14 days) after release and before the radio-tracking started (which was 43 days after her release) she established a new home-range 180 m away. We therefore consider her a dispersing individual.

We used IC-R20 receiver (Icom America Inc.) and 3-element handheld Yagi antenna to locate the animals. The six animals were radio-tracked in rotation with an interval of 30 min between subsequent fixes of the same individual. Radio-tracking of each animal started with an observer checking for its presence in the nest (a single place where it was most frequently encountered) from a distance of 2 m. If the animal was not found in the nest, it was then fixed from a distance of 1–4 m after carefully approaching it. To precisely record the positions of the radio-fixes, we established a geo-referenced 4 m-cell grid of landmarks at the surface, right over the burrow systems actually used by the radio-collared animals before the radio-tracking began. Radio-tracking was performed in three continuous 72-h sessions (3–6 June, 11–14 June and 19–22 June, each session started at 06∶00). After the end of radio-tracking, parts of the burrow systems frequently visited by mole-rats, i.e. the sites, where radio-fixes were clearly concentrated, were uncovered and mapped.

From April to June soil temperatures were recorded every 10 minutes using temperature loggers (Comet System s. r. o., Rožnov pod Radhoštěm, Czech Republic) buried in the study area at a depth of 10, 30 and 60 cm. The depth of 10 cm was chosen to approximate the depth of mole-rat foraging tunnels; the other two depths were selected to illustrate decrease and lag of the fluctuation with the distance from the soil surface.

### Data processing and analysis

To determine daily patterns of activity of the radio-tracked mole-rats, each radio-fix was designated as either inside or outside the nest. According to our previous telemetry studies on mole-rats [4], [20], we estimated the accuracy of our fixes at 0.5 m; thus, all fixes within a 0.5 m radius of the nest were treated as inside the nest.

For each animal, an actogram of the outside-nest activity during three 72-h radio-tracking sessions was created. Based on the actograms, a mean number of activity bouts (periods of time the animal was located outside the nest not interrupted by inside-nest locations) per 24 h and a mean length of an activity bout (the bout lengths were multiples of 30 minutes) were estimated. Subsequently, radio-fixes of each individual from each 72-h radio-tracking session were grouped into 12 2-hour blocks and the proportion of fixes outside the nest was determined for each of the blocks. This data set was used for comparing activity patterns between individual mole-rats and for correlating the activity with environmental variables such as temperature and light.

For each radio-tracked individual separately, and for the whole family (all its radio-collared members pooled), the home range (HR) was defined as a set of cells of a 4m-cell grid (GC) covering radio fixes from all three radio-tracking sessions (432 fixes per individual). For reference purposes, we also computed HR sizes as areas of the minimum convex polygons (MCP). For each radio-tracked member of the family, an exclusive HR was defined as a section of the GC HR which was not found to be utilized by the remaining radio-tracked family members. All HR analyses were performed by ArcGIS 9.3 with the ABODE home range extension [21].

To test the possibility that the existence of the exclusive HR of the four non-breeding family members was just a by-product of the sample size, we used a permutation test. The null hypothesis of the test was that the observed size of the exclusive HR of a particular individual is equal or lower than the corresponding exclusive HR size computed from the data set where coordinate pairs of the outside-nest radio-fixes of the four non-breeders were randomly permutated. The *p*-value for each of the four individuals was calculated as a proportion of cases matching the null hypothesis out of 1000 computations of the exclusive HR size based on the randomly permutated coordinates.

Differences between patterns of outside-nest activity of the radio-tracked individuals were visualized by the principal component analysis (PCA) performed with the software package CANOCO for Windows, version 4.52 [22]. Variables entering the analysis were arcsine transformed and centred (to have zero mean). To evaluate relations between the activity (proportion of outside-nest fixes) and environmental variables we used the Spearman's rank correlation performed with STATISTICA 9 [23]. The environmental factors considered were the soil temperatures in 10, 30 and 60 cm, respectively, and the light-dark cycle with the light hours (06∶00–18∶00 h) marked as 1 and dark hours (18∶00–06∶00 h) marked as 0. Straight distances of the non-breeders' outside-nest fixes from the nest in the light and dark hours were compared by Analysis of variance (ANOVA) in STATISTICA 9 [23] with the tested factors light, individual, and their interaction. Means are given ± SD throughout the text.

## Results

### Spatial patterns of activity

**Figure 1:**
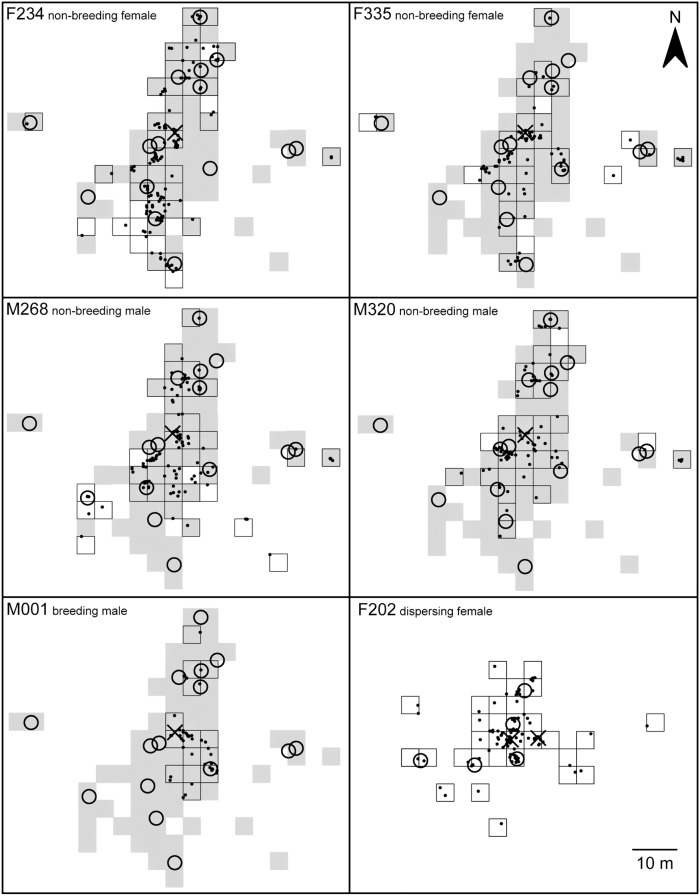
Home ranges of the radio-tracked giant mole-rats. Squares with a black outline represent the GC home range; white squares represent the exclusive home range of a particular individual; the grey area represents GCs of the whole family HR except the GCs already depicted. Dots represent radio-fixes, crosses mark nests and open circles show food sites.

Home ranges (HR) of the radio-tracked mole-rats are depicted in Fig. 1, their sizes, computed both using grid cells (GC) and MCP methods, are presented in Table 1. A mean HR size of a non-breeder of the family was 572±88 m^2^ (GC) which corresponds to 50±8% of the whole family HR. The breeding male M001 had the smallest HR, reaching only 18% of the whole family HR size and he did not use any of its GCs exclusively. The largest HR was that of the female F234, the smallest individual radio-tracked (Table 1). Non-breeders of the family utilized 4–15% of their HRs exclusively (11±5%; Table 1, Fig. 1) which was not significantly more than expected as a by-product of the given sampling intensity (permutation test, *p*>0.05). Nevertheless, in all but one of the non-breeders the observed exclusive HR was larger than the corresponding exclusive HR computed with the coordinate pairs of the outside-nest fixes of the four non-breeders randomly permutated, approaching significance in M268 (*p* = 0.07). All non-breeders' HRs were significantly smaller than corresponding HRs computed with the permutated coordinates (*p*<0.05).

**Table 1:** Home range sizes and proportions of outside-nest fixes of the radio-tracked mole-rats.

Animal	Sex and reproductive/social status	Body mass (g)	Outside-nest radio-fixes (%)	Home range size (m^2^)		Exclusive HR size (m^2^)
				GC	MCP	
M001	Breeding male	454	9.5±2.9	208 (18.3%)	245 (9.6%)	0
M268	Non-breeding male	231	25.2±5.4	608 (53.5%)	1780 (69.7%)	160 (101), *p* = 0.07
M320	Non-breeding male	221	19.7±2.4	512 (45.1%)	1270 (49.7%)	48 (76), *p* = 0.88
F234	Non-breeding female	192	32.2±1.4	688 (60.6%)	2290 (89.6%)	176 (134), *p* = 0.18
F335	Non-breeding female	207	20.4±4.2	496 (43.7%)	2036 (79.7%)	96 (78), *p* = 0.35
Family		20161)		1136	2555	
F202	Dispersing female	220	33.6±2.0	496	1107	

The home ranges (HR) are computed as a set of 4-m grid cells (GC) covering all radio-fixes and as the minimum convex polygon (MCP). Numbers in parentheses signify percentages from the whole family's HR. Exclusive HR size is an area of GCs which contain radio fixes of the focal individual only. Numbers in parentheses are the mean values expected under given sample size if no spatial preferences of the animals occur. The values were obtained based on 1000 permutations with coordinate pairs of the outside-nest fixes of all family non-breeders randomly exchanged. The *p*-values refer to the test of the hypothesis that the size of the exclusive HR is a by-product of the sample size (see Materials and methods for details).

1) Including individuals which were not radio-tracked.

All five radio-collared mole-rats of the family utilized the same nest in the course of the whole study. The nest chamber was located at a depth of 140 cm and it was 26 cm wide and 17 cm high. The dispersing female F202 used two nests successively. She started to use the second nest (located 6 m to the west from the first one; Fig. 1) at the beginning of the third radio-telemetry session. Both nests were relatively small (diameter 16 and 17 cm; hight 13 and 10 cm, respectively) and were located in the depths of 115 and 130 cm, respectively. While being in a nest, the F202 was usually (in 52% of the inside-nest fixes) encountered in the curled-up body position, which contradicts with the family members, whose body position was rarely curled-up (in 10±2% of the inside-nest fixes for non-breeders and in 20% for the breeding male M001).

Besides the nest area, 17 sites with conspicuously concentrated radio-fixes were identified within the HR of the radio-tracked family. Nine of them were foraging areas, where shallow tunnels led to food resources, mainly large tubers of *Dioscorea bulbifera* or *Dioscorea cochleari-apiculata* partly eaten *in situ*. At the remaining eight sites, fresh mounds of soil or freshly backfilled tunnels were found. At some of these “working” sites simultaneous or sequential occurrence of mole-rats had been detected.

### Amounts and temporal patterns of activity

**Figure 2:**
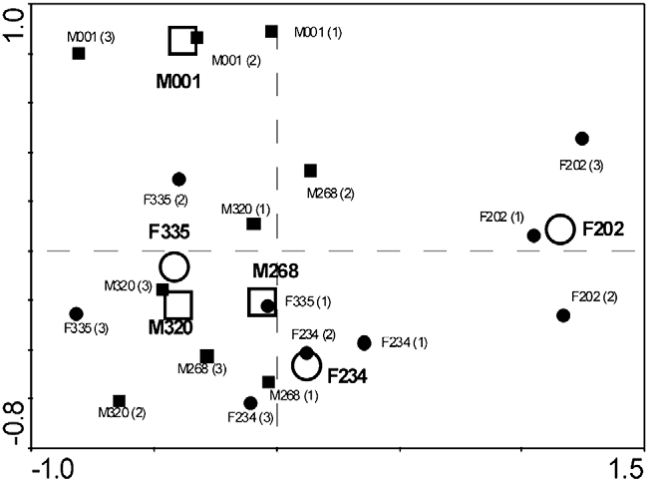
Ordination plot showing similarities between activity patterns of radio-tracked mole-rats. The Principal component analysis; numbers in parentheses indicate particular 72-h radio-tracking sessions, open symbols represent centroids of particular individuals.

**Figure 3:**
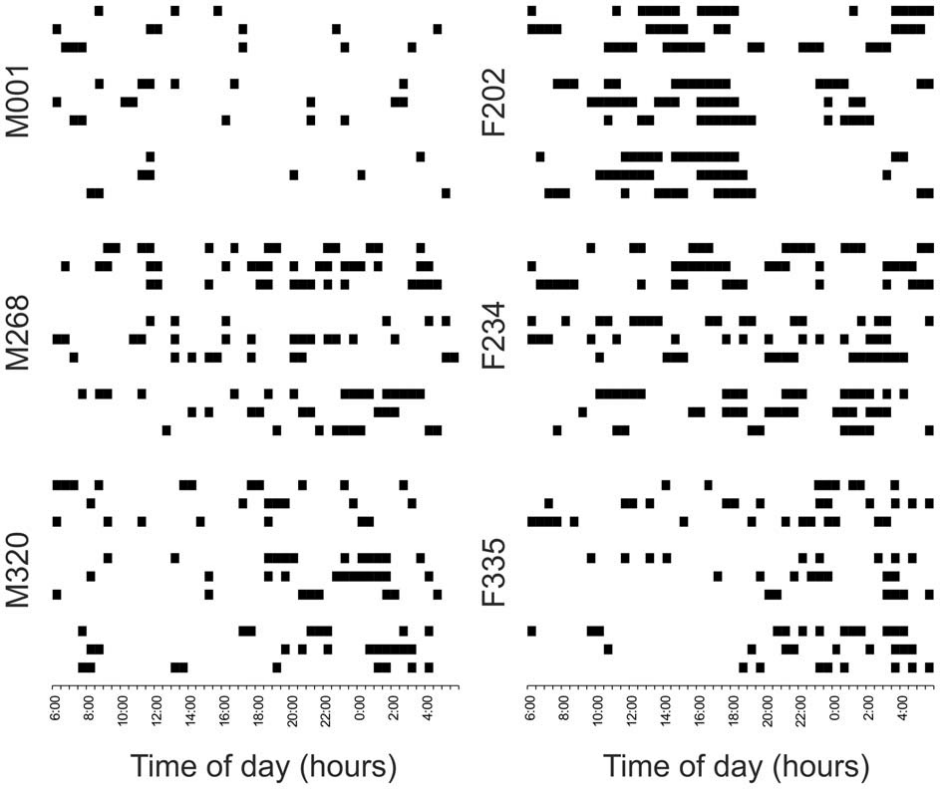
Actograms of the six giant mole-rats radio-tracked during the three continuous 72-h radio-tracking sessions. Dark bars represent bouts of activity (occurrence outside the nest).

Daily amounts of outside-nest activity of the radio-tracked mole-rats are presented in Table 1. The values varied among the individuals, with the breeding male being the least active (3.2 h per day) and the dispersing female F202 the most active (8.0 h per day) individual. The non-breeders were active on average 5.8±1.4 h per day. Temporal patterns of the outside-nest activity were similar in all non-breeders of the family but these patterns differed greatly from those of the breeding male and the dispersing female (Fig. 2). The activity of all mole-rats was polyphasic, with multiple bouts of outside-nest activity during a 24-h cycle (Fig. 3). Non-breeders of the family had 6.5±0.6 bouts of activity per day, which were 54±12 min long. The breeding male had fewer (3.6) bouts of activity per day which were also shorter (38±16 min). The dispersing female F202 had less bouts of activity per day (4.6) than the non-breeders of the family but her bouts were twice as long (109±66 min).

**Figure 4:**
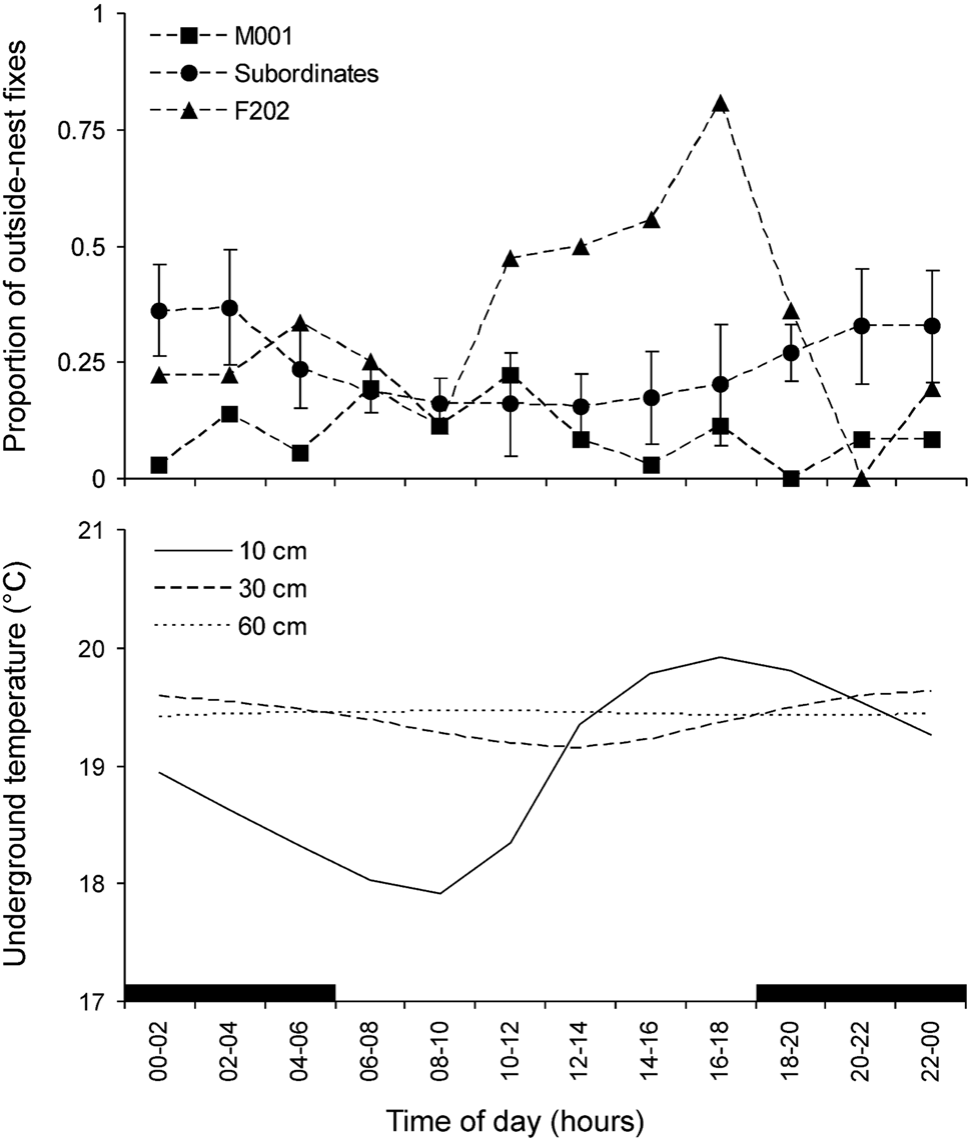
Activity of radio-tracked mole-rats and associated environmental conditions during the 24-h day. Mean ± SD is presented for the four subordinate (non-breeding) family members. Black bars indicate the dark phase of the day.

Individual activity patterns and daily fluctuation of environmental variables are shown in Fig. 4. The correlation matrix of the all variables and individual activity patterns is in Table 2. The activity of the family non-breeders was more concentrated in the dark hours, which is also a period with the maximum temperature at the depth of 30 cm. When active during the dark hours, the non-breeders were located in further distances from the nest than when active during the light hours (F_1, 414_ = 4.5, *p* = 0.03). The effect of individual and interaction between individual and light were not significant (*p* = 0.09 and 0.5, respectively). The activity pattern of the breeding male M001 revealed association with neither environmental variable except the temperature in 60 cm, the fluctuation of which was negligible (Fig. 4). The outside-nest activity of the dispersing female F202 was not significantly correlated with any of the environmental variables, but its peak exactly coincided with the highest daily temperature at the depth of 10 cm (17∶00 h).

**Table 2:** Correlations between activity (proportions of outside-nest fixes) of radio-tracked giant mole-rats and environmental variables set for 12 2-h blocks of the 24-h day.

	M001	F234	M268	M320	F335	Non-breeders	F202	Light/dark cycle	Temperature 10 cm	Temperature 30 cm
F234	−0.17									
M268	−0.34	0.52								
M320	−0.37	0.30	0.53							
F335	−0.10	0.39	0.73*	0.53						
Non-breeders	−0.26	0.67*	0.85*	0.72*	0.85*					
F202	−0.10	−0.01	−0.43	−0.62*	−0.56	−0.49				
Light/dark cycle	0.46	−0.49	–0.85*	−0.67*	−0.88*	−0.87*	0.53			
Temperature 10 cm	−0.49	0.39	0.36	0.03	−0.20	0.16	0.46	−0.10		
Temperature 30 cm	−0.27	0.41	0.85*	0.72*	0.82	0.92*	−0.67*	−0.87*	0.08	
Temperature 60 cm	0.59*	−0.73*	−0.75*	−0.62*	−0.42	−0.77*	0.06	0.68*	–0.66*	–0.66*

For non-breeders the activity in a particular 2-h block was computed as mean over the four animals (F234, M268, M320 and F335).

* *p*<0.05, Spearman correlation coefficient.

## Discussion

Although the biology of African mole-rats is of a great interest to scientists and even to the general public for a long time, efforts to study these animals under natural conditions lag behind. Our study is the first to use radio-telemetry in a study of social mole-rats in more than 20 years. Although the sample size is limited to only six individuals, the study brings several remarkable findings. We proved that temporal and spatial activity pattern differ sharply between a breeding male and non-breeders of the same free-living family group. Unique findings are also those concerning the dispersing female. Since she most likely lived solitarily during the whole period of the study, we obtained a rare chance to describe an activity pattern in a social species which is not actually affected by social interactions, but is still performed under natural conditions.

### Spatial activity patterns

Family groups of social mole-rats occupy extensive burrow systems which are thought to be spatially stable for several years [15], [24]. It should be noted that the area of a burrow system might not correspond with the actual home range, since the animals might not utilize all parts of the burrow system in a given period of time, as it was documented in *Heliophobius argenteocinereus* [20]. So far there were two attempts to delimit HR of a mole-rat family group using radio-telemetry. Lovegrove [10] monitored five individuals of *Fukomys damarensis* from the same family group for three days and calculated their overall HR size (MCP) as 1.3 ha. An even much larger HR of 6.5 ha (recalculated MCP) was used by 20 individuals of *Heterocephalus glaber* monitored sequentially during more than one year by Brett [15]. In the present study the family of the giant mole-rat occupied a relatively small area of 0.26 ha (MCP). All three studies present data on only single mole-rat family groups collected with different sampling lengths, efforts, and designs, which makes the comparison only tentative. Nevertheless, there are at least two reasons to expect that giant mole-rat family groups would have smaller home ranges than the groups of the other two species. First, the giant mole-rat inhabits mesic environments with relatively high food supply [16], [25] whereas the other two species live in arid regions were food density or food biomass is usually low (for recent review see [26]). Second, the other two species can reach higher family group sizes than the giant mole-rat (cf. [7], [16]) which probably signifies a larger available workforce.

The family non-breeders in our study were repeatedly found to occur at some places accompanied by another family mate, or they were found to replace one another during subsequent radio-fixes. At such places, fresh mounds of soil or freshly backfilled tunnels were usually found. This indicates that the animals can cooperate on work tasks. Such cooperation was previously described only in *Heterocephalus glaber* in captivity [27]. On the other hand, all but one non-breeders tended to concentrate their activity to some areas which were partly exclusive (Tab 1, Fig. 1).

### Amounts of activity

We found that non-breeding family members of *F. mechowii* spent only 24% of their time outside the nest. This is the same value as found in *F. damarensis* [10]. On the contrary, the free-living solitary *Heliophobius argenteocinereus* spent as much as 37% of time outside the nest [4]. Considering a principle that the cooperative foraging reduces the risk of starvation [28] the difference is not surprising. The mentioned principle can be interpreted as that under the same conditions the solitary mole-rat must devote larger amount of time to search for food to achieve the same low risk of starvation as a member of a family group.

We observed conspicuous differences in the amount of activity between individuals within the radio-tracked family. The breeding male and the smallest non-breeder radio-tracked (the female F234) stood out from the family, being the least and the most active individuals, respectively (which also corresponds with the smallest and the largest individual HR). Analogous laboratory data on differences in activity between particular members of captive mole-rat families are equivocal. In many studies the least active (or the least working) individuals were breeders (e.g. [12], [29], [30]). On the contrary, no differences in the amount of activity between breeders and non-breeders were found in captive *F. anselli* [14], [31]. In *Cryptomys hottentotus* the breeders were even the most active individuals in a family group [32]. A similar disagreement is characteristic for the relation between relative body mass and the performed activity in non-breeders. Smaller non-breeders usually perform more work (or activity in general) than the larger non-breeders [11], [12], [30], [33] but the opposite was observed in one study on *F. damarensis* [13]. Recently, some light has been shed to this problematic by Scantlebury et al. [34] who investigated daily energy expenditure (DEE) in free-living *F. damarensis* using doubly labelled water (DLW) technique. They found that larger individuals, including breeders, have lower DEE than smaller non-breeders, which is in concordance with our results as well as with the results of most laboratory studies. However, the difference disappeared in the wet season. The breeders and larger non-breeders are thus probably able to mobilize their workforce under some circumstances, for example, when the soil is softened by rains to ease burrowing.

In the radio-tracking study on *F. damarensis* [10] carried out during the rainy season, the largest male, who was considered the breeding male by the author, was similarly active as other family members, but his digging activity (as revealed by the radio signal variation) was probably lower. In the present study, the breeding giant mole-rat male was almost inactive despite the fact that burrowing still occurs during this period [16]. Since he was located mainly around and on the way to places with food resources (Fig. 1) we conclude that he activated predominantly to fulfil his own foraging needs. Our findings therefore suggest that, at least in the dry season, the breeding mole-rat males do not participate in the high-cost work tasks such as burrowing, which is therefore restricted to non-breeders (cf. [11], [35]).

### Temporal activity patterns

Subterranean rodents in captivity usually adjust their daily activity pattern to a given light regime (e.g. [36]–[38]). Nevertheless, the evident retention of the ability to entrain circadian activity rhythms based on the light stimuli does not prove that subterranean rodents universally use the same entrainment mechanism also under natural conditions. In our study, all non-breeding giant mole-rats of the monitored family exhibited similar pattern of outside-nest activity (Fig. 2), where the activity was more concentrated in the dark hours (Fig. 3). However, direct effect of the light stimuli is unlikely if we take into account no aboveground activity and just very infrequent mound building of the radio-tracked mole-rats in the study period.

Circadian activity rhythms can be also entrained using stimuli other than light [39], [40]. For a subterranean mammal a primary candidate would be soil temperature. Like the natural light-dark cycle, the temperature fluctuation follows 24-h periodicity, but its impact could be considered more plausible if the animal encounters light stimuli rarely and irregularly such as in most bathyergids. Several studies show that daily activity rhythms in subterranean rodents can be either entrained or markedly modulated by daily temperature fluctuation [4], [41]–[43]. In the present study, the activity of the family members was correlated with temperature cycles in certain depths (Tab. 2), but the biological significance of the correlations is doubtful. Similarly, Lovegrove [10] found no association between the activity pattern of radio-tracked *F. damarensis* and examined environmental factors, including temperature fluctuation. We propose that temperature-based resetting of circadian clocks still exists in these species, but the activity rhythms are modulated or dissolved by means of other factors and mechanisms. The role of temperature in the mole-rat activity pattern would be necessary to prove using laboratory experiments.

The recorded activity of the giant mole-rats was polyphasic, with multiple bouts of activity per 24-h day separated by periods spent inside the nest. The family non-breeders had on average 6.5 activity bouts per day which were on average 54 min long. This is very similar to the pattern recorded in radio-tracked *F. damarensis* during the hottest period of the year (5.6 bouts per day; 60 min long; [10]). However, during that study, temperatures at the depth of the primary burrows (20–40 cm; [44]) were higher than the upper limit of thermoneutrality zone (TNZ) of the species. The author proposed that the reason why mole-rats engage in such frequent but short bouts of activity is that they avoid hyperthermia during burrowing. In contrast, the giant mole-rats in the present study were radio-tracked during the coldest period of the year with soil temperatures far below the lower limit of the TNZ (TNZ for *F. mechowii* is 29–30°C; [45]). If the cause of such pattern would be analogous to that proposed for *F. damarensis*, with the only difference that the giant mole-rats avoid hypothermia, we would expect (a) that the non-breeding giant mole-rats would perform larger part of their activity in the warmer part of the day in the depth of foraging tunnels, and (b) that the activity performed in short but frequent bouts would be also found in the disperser. Both these predictions were not confirmed by our results. We propose that the similarity in the length of activity bouts and their number per day detected in *F. mechowii* and *F. damarensis* might not be a consequence of a thermoregulatory behaviour, but more likely a result of the similar social behaviour in these two species. The high number of short activity bouts per day could be for example a by-product of the utilization of the communal nest by many individuals (and therefore more frequent disturbance by other family members) or a consequence of cooperation on work tasks.

### Activity of the disperser

Dispersal is a crucial event in the lifetime of an individual. Our data on the dispersing female F202 are the first of its kind in African mole-rats and therefore deserve some discussion although they are limited only to a single individual. We assume that this female left its family (the presence of other mole-rats at the trapping site was clear, because traps and opened tunnels were still getting blocked with soil after capturing the female) and settled in an abandoned part of an existing burrow system. Within six weeks after capture the female F202 was radio-tracked in irregular intervals. The first week she was located close to the place of capture, but the following week she started to move gradually towards her future home range. This would assume she was travelling mostly or entirely underground. Since we did not find many fresh mounds of soil near the radio-fixes, we suppose she used to a large extent an existing tunnel network. The tunnel by which the animal most probably came into the area of her new home range was later found blocked by 1m of soil plug at the edge of her home range. The evidence of the underground dispersal is interesting, since so far the social mole-rats were usually considered to disperse aboveground (cf. [46], [47]). Underground dispersal is probably a much safer strategy but is conditioned by a presence of unused tunnel network in the area or easily workable soil allowing fast burrowing. The *F. mechowii* probably use both ways of dispersal. Kawalika and Burda [19] reported several giant mole-rats captured aboveground, all of them males. It is therefore possible that the strategy of dispersal is sex-specific with females preferring to monopolise a part of an existing burrow network and “wait” for an aboveground dispersing mate to establish a new family. Congruently, Šumbera et al. [16] found two females *F. mechowii* to live separately each at a distant site but still within a single large burrow system occupied by their sister's family group.

We suppose that the female F202 lived solitarily during the whole period of radio-tracking. The reason is that the female usually took curled-up body position when she was in the nest, which contradicts with the radio-tracked family members. The curled-up body position could minimise heat loss via the ventral thermal window [48] and it is the typical resting position for example in the solitary *H. argenteocinereus* [4]. Communally living mole-rats may minimise thermal loses and avoid hypothermia by huddling [48]. During huddling, individuals are usually outstretched in a group with their bodies in a more or less horizontal position.

The dispersing female exhibited a single peak of activity per day, which coincided with the temperature maximum in the surface layer of soil, where foraging tunnels occur (Fig. 4). Given that the study took place in the coldest period of year, we propose that the female preferred to be active in the part of the day with the most comfortable temperatures for work in the surface tunnels. Her daily activity was concentrated into fewer activity bouts, which possibly reflect the absence of social cues to trigger activity. As a whole, the behaviour of this individual indicates that daily amounts of activity and activity patterns are flexible in social mole-rats and can be adjusted to the actual environmental and social conditions.
